# Suitability of transbronchial needle aspiration for genotyping peripheral pulmonary tumors

**DOI:** 10.3389/fmed.2022.1087028

**Published:** 2023-01-12

**Authors:** Lina Zuccatosta, Letizia Latini, Beatrice Belleggia, Francesca Gonnelli, Francesca Barbisan, Gaia Goteri, Stefano Gasparini, Antonio Marchetti

**Affiliations:** ^1^Pulmonary Diseases Unit, Azienda “Ospedali Riuniti”, Ancona, Italy; ^2^Department of Biomedical Sciences and Public Health, Polytechnic University of Marche Region, Ancona, Italy; ^3^Pathological Anatomy Institute, Polytechnic University of Marche Region, Ancona, Italy; ^4^Laboratory of Diagnostic Molecular Oncology, Center for Advanced Study and Technology (CAST), University of Chieti, Chieti, Italy

**Keywords:** peripheral pulmonary tumors, bronchoscopy, transbronchial needle aspiration, genotype, mutational status

## Abstract

**Background:**

Transbronchial needle aspiration (TBNA) is a sampling tool that has demonstrated a higher accuracy in the diagnosis of peripheral pulmonary lesions (PPL) compared to other techniques. However, there are no studies investigating the value of TBNA in defining the genotype of peripheral lung cancer.

**Objective:**

To evaluate the accuracy of TBNA in defining the molecular characteristics of peripheral lung cancer.

**Methods:**

Consecutive patients who underwent TBNA for the diagnosis of a PPL at the Pulmonary Unit of the Azienda Ospedali Riuniti of Ancona (Italy) between January 2020 and September 2022 were included in the study. TBNA was performed under fluoroscopic guidance and the additional support of an ultrasound miniprobe, with an ultrathin bronchoscope with a flexible 21G needle. Samples were smeared on glass slides for cytological evaluation and flushed in 10% neutral-buffered formalin for cell-blocks.

**Results:**

154 patients were enrolled:55 were diagnosed with adenocarcinoma and 21 with squamous cell carcinoma. TBNA correctly diagnosed 43/55 (78.2%) patients with adenocarcinoma and 17/21 (81.0%) patients with squamous cell carcinoma, with a sensitivity of 77.5%. Complete genotyping for guiding targeted therapies was obtained in 52 patients (86.6%).

**Conclusions:**

TBNA is a valid tool for the diagnosis of PPL, allowing a correct diagnosis and a complete genotyping of the tumors in a considerable proportion of patients.

## Introduction

The transbronchial approach is widely used for the diagnosis of peripheral pulmonary lesions (PPL). It can be performed with different guidance systems and with different sampling instruments. Over the last decades, in addition to the traditional fluoroscopy, new guidance systems have been developed (radial ultrasound mini probes, electromagnetic navigation bronchoscopy, virtual bronchoscopy, cone beam CT, and robotic bronchoscopy), aiming to improve diagnostic yield, especially for small lesions (1).

In addition to the guidance system, several sampling tools (forceps biopsy, flexible needle, curettes, brushing, and cryoprobe) can be used to obtain adequate diagnostic material (2).

Although a standardized approach is still lacking and the use of one or more sampling instrument generally depends on local availability and operators' experience, there is strong evidence that transbronchial needle aspiration (TBNA) is the sampling tool with the highest diagnostic yield in the transbronchial approach to PPL (3–5). The ability of the needle to penetrate the lesion even if it does not involve the endobronchial surface is the main reason for greater results of TBNA (2, 3). Furthermore, there is good evidence that the diagnostic accuracy of TBNA in the diagnosis of PPL is increased if more than one sampling instrument is used (6, 7).

However, in the era of tailored therapy of lung cancer, histological diagnosis is not enough to guide the treatment and a molecular evaluation, including programmed cell death ligand-1 (PD-L1), is highly recommended.

The possibility of genotyping tumors with samples obtained by needle aspiration has been confirmed by several studies (8–12). However, these studies have been performed with samples obtained by endobronchial ultrasound-guided TBNA (EBUS-TBNA) on mediastinal lymph nodes.

To the best of our knowledge, there are no large studies describing the yield of TBNA in molecular profiling of peripheral lung cancer.

Therefore, the main aim of the present study was to evaluate the accuracy of TBNA in genotyping peripheral lung cancer. The secondary outcome was to evaluate whether the addition of forceps biopsy to TBNA improved the yield and the adequacy of the specimens for molecular diagnosis.

## Materials and methods

### Subjects

This is a single-center retrospective study performed at the Pulmonary Diseases Unit of the Ancona Hospital (Italy) between January 2020 and September 2022.Consecutive patients who underwent TBNA for diagnosing PPL were recruited. A PPL was defined as a lung lesion not visible through the flexible bronchoscope.

### Bronchoscopic procedure and sample management

Bronchoscopic procedures were performed under general anesthesia through a laryngeal mask using an ultrathin bronchoscope (Olympus BF-MP190F). After careful examination of the bronchial tree, the bronchoscope was inserted into the tributary subsegmental bronchus of the lesion, previously identified by CT scan. Under fluoroscopic guidance, the bronchoscope was advanced as close as possible to the lesion. Then, a radial ultrasound miniprobe (r-EBUS) was used to localize the lesion together with fluoroscopic guidance. Once the lesion was visualized by rEBUS, the miniprobe was withdrawn and a flexible 21G needle (PeriView Flex, Olympus) was inserted under fluoroscopic control in the same position. After the correct position of the needle was reached, the needle was advanced and moved within the lesion while a suction was applied for 5–8 s with a 20 mL syringe connected to the proximal end of the needle sheath. The needle was then withdrawn. Four needle passes were performed for each lesion.

The material obtained by the first needle pass was pushed on clean glass slides, rapidly smeared and fixed in 95% ethanol. One glass was utilized for rapid on-site cytological evaluation (ROSE) using rapid stain method Hemacolor-Merck and evaluated by a cytopathologist or a pulmonologist trained in cytopathology (13). If ROSE was positive for diagnostic cells, three additional passes with the needle were performed and the material was flushed in 10% neutral-buffered formalin for cell-block. If ROSE did not demonstrate diagnostic cells, the rEBUS was repeated to better identify the lesion, and potentially changing the subsegmental bronchus approached. Regardless of whether or not the second ROSE results, three needle passes for cell block were performed.

If the ROSE was positive for atypical cells but the material was judged poor due to the presence of necrosis or few diagnostic cells, three additional samples with biopsy forceps (TBPB) (Olympus miniforceps FB-456D) were obtained.

### Histological evaluation and molecular testing

Cell blocks were fixed in formalin for 6–48 h and embedded in paraffin. Cytological glasses were stained with Papanicolaou, while cell block sections with hematoxylin-eosin. Both kinds of material were used for morphological evaluation: In addition, immunohistochemical staining was performed on cell block sections for the final histological diagnosis.

In case of lung primary adenocarcinoma, cytological smears were used for DNA extraction (QIA amp DNA mini kit). Smears were considered adequate if at least 500 neoplastic cells were present with a tumor vital cellularity equal or above 50%.

Mutational analysis of the 10 genes commonly involved in NSCLC (EGFR, KRAS, BRAF, PIK3CA, NRAS, ALK, ERBB2, DDR2, MAP2K1 and RET) was performed by MALDI-TOF Mass Spectrometry (MassARRAY, Agena Bioscience) using the Myriapod Lung Status Kit (Diatech Pharmacogenetics). Cell blocks were utilized for immunohistochemical predictive markers ALK (D5F3 CDx assay on platform BenchMark ULTRA Ventana Medical Systems Inc.), ROS1 (Ventana SP384 Rabbit Monoclonal Primary Antibody on platform BenchMark ULTRA Ventana Medical Systems Inc.), PD-L1 (PD-L1 IHC 22C3 pharmDx for Autostainer Link 48 - Agilent). MALDI-TOF is a technology that allows multiplexed genotyping; it has been adopted in routine diagnostics as a sensitive, reliable, fast, and cost-effective method. It detects targetable mutations, and it is quite effective even in low-quality samples. Furthermore, our panel (Myriapod Lung Status Kit) is able to detect the most common driver mutations in NSCLC. Cell blocks sections were considered adequate if at least 100 neoplastic cells were present.

### Statistical analysis

Descriptive data were presented as means, frequencies and percentages as appropriate. The sensitivity for TBNA and TBPB was evaluated as the ratio between correct diagnoses and the sum of true diagnoses and false negatives. The adequacy for genotyping was assessed as percentage of patients in whom a complete molecular panel was possible out of the total number of patients diagnosed with primary lung cancer by transbronchial approach.

### Ethical aspects

This retrospective study was approved by the Ethical Committee of Marche Region (343/2022).

Due to the retrospective nature of the study and since data were de-identified, the need for informed consent was waived.

## Results

During the study period 154 consecutive patients (M = 101; F = 53; mean age = 69.8 yrs; min = 26, max = 93) underwent bronchoscopic approach to PPL with TBNA and TBPB under fluoroscopic and rEBUS guidance at our Institution. There were 88 smokers, 32 former smokers and 34 non-smokers.

The mean diameter of the PPL was 37 mm ± 13 SD (range: 13–60) (Table 1).

**Table 1:** Characteristics of the patients.

**Patients *n***	**154**
Mean age (range) years	69.8 (26–93)
Sex (M/F)	101/53
**Tobacco**	
- Non smokers *n* (%) - Current smokers *n* (%) - Former smokers *n* (%)	34 (22.1) 88 (57.1) 32 (20.8)
Lesion size (mm): mean ± SD (min, max)	37 ± 13 (13–60)

The final diagnosis for each patient enrolled in the study is reported in Table 2. In particular, 98 patients were diagnosed with a malignant neoplasm, 43 with a benign neoplasm, and in 13 patients a definite diagnosis was not available and thus they were not included in the analyses.

**Table 2:** Definitive diagnosis and diagnosis obtained by TBNA in 154 patients with PPL.

**Diagnosis**	**Final diagnosis *n* (% of total)**	**Diagnosis obtained by TBNA *n* (diagnostic yield % for each pathology)**
**Malignant lesions**	98 (63.6)	76 (77.5)
Primary lung cancer - Adenocarcinoma - Squamous cell - Small cell	55 (35.7) 21 (13.6) 3 (1.9)	43 (78.1) 17 (80.9) 3 (100)
Metastases	15 (9.7)	10 (66.6)
Carcinoid	4 (2.6)	3 (75)
**Benign lesions**	43 (27.9)	17 (39.5)
Hamartoma	5 (3.2)	2 (40)
Inflammatory lesions - Pneumonia - Abscess - OP - Granulomas - Fibrotic lesion - Lipoid pneumonia - Other	38 (24.6) 11 7 6 6 3 2 3	15 (39.4) 6 6 0 2 0 0 1
**Lost to follow-up**	13 (8.4%)	No diagnosis

TBNA allowed a correct diagnosis in 93 patients with a diagnostic yield of 60.3%.

Regarding the 98 malignant lesions, TBNA provided a diagnosis in 76 cases (Table 3).

Table 3Molecular characterization of 39 patients with primary lung adenocarcinoma obtained by TBNA material.

**Table:** 

	**1**	**2**	**3**	**4**	**5**	**6**	**7**	**8**	**9**	**10**	**11**	**12**	**13**	**14**
Sex	F	M	M	M	M	M	M	M	F	F	M	M	M	F
N.neopl.cell	1,000	1,000	1,000	1,000	1,000	1,000	1,000	1,000	800	1,000	1,000	1,000	1,000	800
Vital cells %	70	70	80	70	80	80	80	80	60	70	60	80	80	50
Extracted DNA ng/μl	52.7	23.0	57.4	30.4	137.3	140.6	52.0	110.6	8.0	100.38	139.99	220.43	28.3	4.80
EGFR	WT	WT	WT	WT	WT	WT	WT	WT	WT	WT	WT	WT	WT	WT
KRAS	WT				WT	WT		WT		WT	WT	WT		
BRAF	WT	WT	WT	WT	WT	WT	WT	WT	WT	WT	WT	WT	WT	WT
NRAS	WT	WT	WT	WT	WT	WT	WT	WT	WT	WT	WT	WT	WT	WT
PIK3CA	WT	WT	WT	WT	WT	WT	WT	WT	WT	WT	WT	WT	WT	WT
ALK	WT	WT	WT	WT	WT	WT	WT	WT	WT	WT	WT	WT	WT	WT
ERBB2	WT	WT	WT	WT	WT	WT	WT	WT	WT	WT	WT	WT	WT	WT
DDR2	WT	WT	WT	WT	WT	WT	WT	WT	WT	WT	WT	WT	WT	WT
MAP2K1	WT	WT	WT	WT	WT	WT	WT	WT	WT	WT	WT	WT	WT	WT
RET	WT	WT	WT	WT	WT	WT	WT	WT	WT	WT	WT	WT	WT	WT
ALK (IHC)	Neg	Neg	Neg	Neg	Neg	Neg	Neg	Neg	Neg	Neg	Neg	Neg	Neg	Neg
ROS (IHC)	Neg	Neg	Neg	Neg	Neg	Neg	Neg	Neg	Neg	Neg	Neg	Neg	Neg	Neg
PDL1	1%	60%	10%	Neg	Neg	1%	30%	60%	Neg	Neg	1%	20%	30%	60%

**Table:** 

	**15**	**16**	**17**	**18**	**19**	**20**	**21**	**22**	**23**	**24**	**25**	**26**	**27**	**28**	**29**
Sex	F	M	M	M	F	F	M	M	F	M	M	M	F	M	M
N.neopl.cell	1,000	800	1,000	800	800	1,000	1,000	1,000	1,000	1,000	1,000	1,000	1,000	1,000	1,000
Vital cells %	70	60	80	70	70	80	60	90	80	60	80	70	60	50%	70%
Extracted DNA ng/μl	8.7	14.0	63.0	16.9	2.66	14.5	30.3	270.9	68.02	89.5	92.0	664.4	125.5	5.9	21.3
EGFR		WT	WT	WT		WT	WT	WT	WT		WT	WT		WT	
KRAS	WT	WT	WT		WT		WT	WT	WT	WT	WT		WT	WT	WT
BRAF	WT	WT	WT	WT	WT	WT	WT	WT	WT	WT	WT	WT	WT	WT	WT
NRAS	WT	WT	WT	WT	WT	WT	WT	WT	WT	WT	WT	WT	WT	WT	WT
PIK3CA	WT	WT	WT	WT		WT	WT	WT	WT	WT	WT		WT	WT	WT
ALK	WT	WT	WT	WT	WT	WT	WT	WT	WT	WT	WT	WT	WT	WT	WT
ERBB2	WT	WT	WT	WT	WT	WT	WT	WT	WT	WT	WT	WT	WT	WT	WT
DDR2	WT	WT	WT	WT	WT	WT	WT	WT	WT	WT	WT	WT	WT	WT	WT
MAP2K1	WT	WT	WT	WT	WT	WT	WT	WT	WT	WT	WT	WT	WT	WT	WT
RET	WT	WT	WT	WT	WT	WT	WT	WT	WT	WT	WT	WT	WT	WT	WT
ALK (IHC)	Neg	Neg	Neg	Neg	Neg	Neg	Neg	Neg	Neg	Neg	Neg	Neg	Neg	Neg	Neg
ROS (IHC)	Neg	Neg	Neg	Neg	Neg	Neg	Neg	Neg	Neg	Neg	Neg	Neg	Neg	Neg	Neg
PDL1	Neg	Neg	Neg	40%	Neg	1%	70%	5%	1%	Neg	10%	Neg	Neg	10%	1%

**Table:** 

	**30**	**31**	**32**	**33**	**34**	**34**	**35**	**36**	**38**	**39**
Sex	F	F	M	F	M	M	M	M	M	F
N.neopl.cell	1,000	1,000	1,000	1,000	1,000	1,000	1,000	1,000	1,000	1,000
Vital cells %	80	60	80	80	80	70	70	70	50	70
Extracted DNA ng/μl	20.5	49.1	31.0	277.2	17.6	130.1	23.1	36.7	48.0	52.7
EGFR	WT		WT		WT	WT	WT	WT	WT	
KRAS		WT		WT	WT	WT	WT	WT	WT	WT
BRAF	WT	WT	WT	WT	WT	WT	WT	WT	WT	WT
NRAS	WT	WT	WT	WT	WT	WT	WT	WT	WT	WT
PIK3CA	WT	WT	WT	WT	WT	WT	WT	WT		WT
ALK	WT	WT	WT	WT	WT	WT	WT	WT	WT	WT
ERBB2	WT	WT	WT	WT	WT	WT	WT	WT	WT	WT
DDR2	WT	WT	WT	WT	WT	WT	WT	WT	WT	WT
MAP2K1	WT	WT	WT	WT	WT	WT	WT	WT	WT	WT
RET	WT	WT	WT	WT	WT	WT	WT	WT	WT	WT
ALK (IHC)	Neg	Neg	Neg	Neg	Neg	Neg	Neg	Neg	Neg	Neg
ROS (IHC)	Neg	Neg	Neg	Neg	Neg	Neg	Neg	Neg	Neg	Neg
PDL1	Neg	Neg	30%	2%	20%	5%	1%	Neg	2%	1%

Gray cells are indicative of mutations. ICH, Immunohistochemistry; WT, Wild type.

TBNA sensitivity and negative predictive value (NPV) for malignancy (including cases of carcinoid) were respectively 77.5% and 66.1%.

Among the 76 patients with primary non-small cell lung cancer (55 adenocarcinomas and 21 squamous cell carcinoma), 27 cases (35.5%) were in stage IV for presence of distant metastases.

The definition of tumor genotype was performed on the 60 patients with diagnosis of primary lung adenocarcinoma (*n* = 43) or squamous cell carcinoma (*n* = 17). The TBNA material was adequate for genotyping in 52/60 patients (86.6%), (39/43, 90.7% adenocarcinoma, 13/17, 76.5% squamous cell carcinoma). In 8/60 patients, the specimens were adequate to provide a diagnosis but, due to necrotic tissue or small number of vital neoplastic cells, the definition of genotype was not possible.

The use of forceps biopsy in addition to TBNA was performed in 10 cases, based on the indication of the ROSE that showed poor diagnostic material. In 4/8 patients with inadequate TBNA material, the genotyping was possible using biopsy specimens. The number of patients for which genotyping was possible combining TBNA and TBPB materials was 56/60 (93.3%). The number of neoplastic cells on TBNA samples obtained from patients affected by adenocarcinoma was >1,000 in 33 cases, >800 in 2, > 500 in 4. For the evaluation of PD-L1 the number of neoplastic cells evaluated was > 100 in all 52 cases. The mean concentration of extracted DNA in patients with adenocarcinoma was 81.27 ng/μl (min: 4.80 ng/μl; max: 664.40). The molecular characterization of the 39 patients affected by adenocarcinoma is reported in Table 3. Table 4 shows the results of PD-L1 assessment in 13 patients with squamous cell carcinoma.

**Table 4:** PD-L1 expression evaluated on TBNA material in 13 patients affected by squamous cell carcinoma.

	**1**	**2**	**3**	**4**	**5**	**6**	**7**	**8**	**9**	**10**	**11**	**12**	**13**
Sex	M	M	M	F	M	F	M	M	M	M	M	F	M
*N* neoplastic cells	>100	>100	>100	>100	>100	>100	>100	>100	>100	>100	>100	>100	>100
PD-L1	10%	10%	Neg	30%	Neg	10%	5%	1%	60%	40%	20%	30%	30%

The mutations identified in patients affected by adenocarcinoma were: mutation of KRAS in 12 patients (exon 2, codon 11 in 12 and exon 3, codon 61 in 1). EGFR alterations were characterized by deletion of exon 19 in 3 patients, by mutation in exon 21 in 2, mutations of exon 20 in 1, mutations in exons 20 and 21 in 1 and mutations in exons 19 and 21 in 1. In 3 patients a mutation of exon 21 of PIK3CA was found. We did not identify patients with ALK or ROS1 translocation.

The TBNA/TBNB procedures were safe, as only one pneumothorax requiring chest tube insertion was reported (0.6%).

## Discussion

Among the different sampling instruments employed for the transbronchial approach to PPL, TBNA provides the highest diagnostic yield (2–6). In the meta-analysis by Mondoni et al. (5), TBNA showed a better diagnostic yield (60%) than forceps biopsy (45%). However, this meta-analysis demonstrated a high heterogeneity of results, as a consequence of the size of the lesion (55% for PPL ≤3 cm, 81% for PPL>3 cm), the nature of the lesion (55% for malignant PPL; 17% for benign PPL), the presence of bronchus sign (70% if bronchus sign was present; 51 % if bronchus sign was absent), and the use of ROSE (62 % if ROSE was performed; 51% when ROSE was not carried out). The major advantage of TBNA in sampling PPL is the ability of the needle to penetrate the lesion even if it does not involve the endobronchial surface or if it is located adjacent to a bronchus or to a bronchiolar spur. In these conditions, the needle can penetrate the bronchial wall and reach the target. The use of an ultrathin bronchoscope can further increase the diagnostic performance, bringing the bronchoscope closer to the lesion and allowing the needle to pass through the bronchial wall by flexing the tip of the instrument (3). However, TBNA is a sampling instrument that can also be used with a standard flexible bronchoscope as well as with all available guidance systems (electromagnetic navigation bronchoscopy, robotic bronchoscopy, virtual bronchoscopy and cone beam CT).

Even though the evaluation of TBNA sensitivity was not the aim of this study, our data showed a TBNA overall diagnostic yield of 60.3% and a sensitivity for malignant lesion of 77.5%, that is comparable with the results of above-mentioned meta-analysis (5), considering the mean size of the lesions and the use of the ROSE.

However, in the era of targeted therapy for lung cancer, the diagnostic yield alone cannot be considered a sufficient criteria to validate a sampling technique. It is also necessary that the sample is adequate for a molecular genotyping of the tumor as guidelines recommend acquisition of adequate material for molecular tests during the initial work-up of lung cancer (14). Even if most cases of peripheral lung cancer are suitable for surgery, it is not rare that a peripheral cancer is not appropriate for surgical resection due to presence of distal metastases, major comorbidities or impaired cardiorespiratory function. For this reason, it is important to have a sampling method that reliably allows the molecular characterization of the tumor in order to optimize the therapeutic management.

Tumor genotyping using material obtained from needle aspiration techniques has been described in several papers. Xie et al. (8) evaluated sampling from EBUS-TBNA performed on 85 patients with non-small cell lung cancer and were able to genotyping tumors in 77 patients (90.6%), using both conventional gene tests (polymerase chain reaction-PCR, immunohistochemistry and fluorescence *in situ* hybridization-FISH) and next-generation sequencing (NGS). In a study on 114 patients affected by adenocarcinoma or not otherwise specified lung cancer, Cicek et al. (15) found adequate material for EGFR mutation in 88.6%, for ALK and ROS1 rearrangement, respectively in 93.8% and 91.8%. In a large multicenter study on 774 patients, Navani *N* et al. reported that EGFR mutation analysis was possible in 90% of cases (9). Martin-Deleon et al. evaluated EBUS-TBNA samples for several biomarkers on 42 patients affected by non-small cell lung cancer and reported 92.9% successfully genotyped by next generation sequencing, 95.2% by nCounter and 100% by immunohistochemistry (10). Other studies have also demonstrated that EBUS-TBNA samples is able to provide enough material for PD-L1 determination in 85-90% of specimens (14, 16, 17).

However, all these studies were performed mainly on specimens obtained by EBUS-TBNA. In the paper by Stoy et al. (17), PD-L1 was evaluated on different cytology-needle based bronchoscopic sampling techniques, but only in 2 patients TBNA was performed on PPLs.

The present study aimed for the first time to evaluate the role of TBNA in genotyping lung peripheral tumors on a large number of patients.

Our data showed that the specimens obtained by TBNA of peripheral lesions provided adequate material for full molecular profile of the tumor for current therapies (including PD-L1 expression) in 86.6% of patients with non-small cell lung cancer. It is important to underline that, to obtain this result, an essential step is the correct handling of the sample. In our series, the smeared cytology and the cell block were both evaluated to provide a complete genotyping of the tumor. Cytological smears were used for DNA extraction and cell block for immunohistochemical staining to define the histotype and for predictive markers such as ALK, ROS1 and PD-L1. The integration of smeared cytology and cell block material to optimize the capability of genotyping the tumor has been already reported in the literature (18, 19). Fielding et al. (20) achieved high concordance rates in detecting mutations between smeared cytology and cell block and showed that DNA extracted from smears has higher rates of mutations than DNA obtained by cell block. Robin M et al. (21), using both cytology and histology samples, were able to genotyping the tumor in 69.1% and to evaluate PD-L1 expression in 94% of 331 patients who underwent transbronchial approach to lung tumor with the guidance of rEBUS or electromagnetic navigation. However, in that study the cytological material was obtained only by brushing and catheter rinse. The smaller amount of cytological material obtained by these sampling techniques in comparison to TBNA could explain the lower percentage of patients in whom genotyping was possible in comparison to our results.

The diagnostic yield of transbronchial approach to PPL increased if more than one sampling instrument is used (2, 7). In a previous study performed at our Institution, we performed TBNA and TBPB in the diagnosis of patients with PPL and demonstrated that, utilizing both sampling instruments. sensitivity increased from 69.3% (TBNA alone) to 75.4% (TBNA+TBPB). In the present study we performed biopsy forceps in addition to TBNA in 10 patients, but only when ROSE showed poor material at the first needle pass. The reason why TBPB was not used in all cases was to reduce the cost of the procedure, since the mini forceps that must be employed with an ultrathin bronchoscope are disposable. It would be interesting to carry out a further randomized study to verify whether the systematic use of forceps biopsy can improve the results compared to the use of forceps based on the results of ROSE. However, in our series, the specimens obtained by forceps biopsy allowed to genotyping 4 more patients, and thus increasing the adequacy of the sample from 86.6 to 93.3%. The value of ROSE in increasing the possibility of genotyping the tumor sampled by EBUS-TBNA, was demonstrated in a randomized trial by Trisolini et al. (22). In this study 98 patients underwent EBUS-TBNA with ROSE and 99 without ROSE. In the ROSE group genotyping was completed in 90.8 vs. 80.3% in the non-ROSE group.

The high prevalence of KRAS mutation in our patients with adenocarcinoma (30.7%) is comparable to what is reported in the literature for Caucasian patients (23). The EGFR mutations were found in 8 patients (20.5%), which is a bit higher than that reported for Western Europe patients (10–15%) (24). This could be related to the higher percentage of female in our population (33.3%).

In the last years, the use of cryoprobe has been proposed to increase the amount of tissue obtained by the transbronchial approach to peripheral lung tumors, thus improving the possibility of genotyping the tumor. It has been proved that cryobiopsy can obtain tissue even in tumor adjacent to the airways. The better sensitivity and the greater specimens of cryobiopsy in comparison with conventional forceps biopsy have been demonstrated in several studies (25–29). However, no studies have compared cryobiopsy vs. TBNA. It would be desirable that randomized trials will be carried out to evaluate the diagnostic yield, the sample adequacy for genotyping and the safety of the two techniques.

Some limitations of this paper should be acknowledged. The study was performed at a single Institution with a long experience in the diagnosis of PPL with TBNA and in the use of ROSE (6). In addition, the molecular analysis performed in this study was limited to a multiplex test for the detection of hot spot mutations using an extremely sensitive MALDI-TOF Mass Spectrometry assay. However, the sensitivity of other commercially available multiplex assays, including next generation sequencing (NGS), has increased in the last years and should now be comparable, particularly when genomic panel for hotspot lesions are used.

In conclusion, our study confirms the validity of TBNA as a valuable tool in the diagnostic approach to PPL. Furthermore, this is the first report that demonstrates the value of TBNA also in genotyping peripheral pulmonary tumors. The proper handling of the specimens with the integration of smeared cytology and cell block is an essential step to optimize the results. The use of ROSE appears to improve the diagnostic yield by revealing the need to use other sampling tools if the material obtained by TBNA appears to be of low quality due to the presence of few neoplastic cells or necrosis.

## Data availability statement

The raw data supporting the conclusions of this article will be made available by the authors, without undue reservation.

## Ethics statement

The studies involving human participants were reviewed and approved by Comitato Etico delle Marche (343/2022). The patients/participants provided their written informed consent to participate in this study.

## Author contributions

LZ, FB, SG, and AM contributed substantially to the conception, design of the work, and drafted the work. LZ, LL, BB, FG, FB, and GG contributed to the collection of data. LZ, LL, BB, FG, FB, GG, SG, and AM contributed to the interpretation of data and revisions. All authors have reviewed and approved the final version of the manuscript.
